# Hydatid Disease

**Published:** 1895-03

**Authors:** 


*Hydatid Disease.* Vol. II. By the late JOHN DAVIES THOMAS, M.D. Pp. xii., 166. Sydney: L. BRUCK. 1894.

This book is a very valuable contribution to our knowledge of hydatid disease. It is written by a medical man who practised in Australia, where the disease is so common, and occurs not infrequently in those rarer forms which we seldom see in this country. In 1884 Dr. Thomas published the first volume, the title of which was *Hydatid Disease, with Special Reference to its Prevalence in Australia.* Unfortunately his health broke down before he had completed the second volume. But it has been edited by Dr. Lendon, his former partner, who has not added to or altered the text.

This volume is clinical in its scope, and deals with hydatid disease affecting various organs and tissues. The chapter on "Hydatid Disease of the Lungs" is particularly valuable, as the disease in those organs is rare in this country, but by no means so in Australia. The last portion of the work is devoted to the consideration of the various methods of treating hydatids by operation. The volume is evidently the result of very laborious study and careful clinical observation.

